# Preliminary Assessment of Thermal and Mechanical Properties of a Graphene-Rich Carbon Coating on 3003-H14 Aluminum Alloy for Potential Anti-Icing Applications

**DOI:** 10.3390/ma19061150

**Published:** 2026-03-16

**Authors:** Abdallah Almomani, Mu’nis Alkhasawneh, Mohammed A. Almomani, Muath A. Bani-Hani

**Affiliations:** 1 Aeronautical Engineering Department, Faculty of Engineering, Jordan University of Science and Technology, P.O. Box 3030, Irbid 22110, Jordan; 2 Industrial Engineering Department, Faculty of Engineering, Jordan University of Science and Technology, P.O. Box 3030, Irbid 22110, Jordan

**Keywords:** anti-icing, de-icing, 3003-H14 aluminum alloy, aircraft engine components, graphene nano-coating

## Abstract

Icing poses significant operational and safety risks in aviation, especially for engine components such as cowls and baffles. This study explores the potential of a chemically exfoliated graphene-rich carbon platelet epoxy coating to improve the anti-icing and de-icing performance of 3003-H14 aluminum alloy, which is widely used in such applications. Chemically exfoliated graphite was incorporated into an epoxy resin, then applied to aluminum substrates. Characterization of the coated samples revealed ~30% improvement in surface Vickers hardness (HV) (HV 75.6 ± 1.15 vs. HV average of 98.3 ± 1.5) and enhanced thermal dissipation, with coated surfaces cooling from 104 °C to 22 °C in 530 s compared to 870 s for uncoated samples. While anti-icing performance was not directly evaluated, the observed improvements in thermal dissipation and surface hardness suggest that chemically exfoliated graphene-rich carbon platelet coatings could be promising for passive anti-icing applications. The literature suggests that graphene coating improves hydrophobicity, reducing ice adhesion and delaying nucleation due to its low surface energy and nanoscale roughness, thereby supporting potential passive anti-icing functionality for aircraft engine components. SEM analysis confirmed a uniform, compact coating layer. These preliminary findings indicate that chemically exfoliated graphene-rich carbon platelet coatings can deliver multifunctional performance—mechanical, thermal, and surface—making them promising candidates for passive anti-icing/de-icing solutions in engine components where conventional systems are ineffective.

## 1. Introduction

Icing is a critical weather-related concern in the aviation industry, with potentially severe consequences for aircraft operations [1,2,3]. In cold and humid environments, ice accretion on aircraft surfaces can lead to serious flight hazards, including increased weight, aerodynamic imbalance, and reduced controllability [4]. These conditions often result in flight delays, cancellations, and increased fuel consumption. Therefore, effective de-icing (removal of ice) and anti-icing (prevention of ice formation) strategies are essential for maintaining operational safety and efficiency in modern aviation.

Ice typically forms when atmospheric moisture freezes upon contact with cold aircraft surfaces—particularly flight-critical control elements such as wings, tails, and ailerons. Ice accumulation alters aerodynamic properties by increasing surface roughness and weight, thereby reducing lift and control. This change in aerodynamic profile poses serious safety risks, particularly during takeoff and landing [5]. The 24th International Congress of the Aeronautical Sciences (2004) stressed the urgency of advancing anti-/de-icing technologies to address these risks, and Z. Goraj [1] emphasized that mitigating aerodynamic surface contamination remains central to aviation safety strategies.

However, most existing studies and technologies focus on aerodynamic surfaces such as wings, while engine cowls and baffles—components highly susceptible to icing—remain largely unaddressed. This represents a significant gap in both research and application.

Several technologies have been developed for in-flight and ground-based de-icing systems. Pneumatic systems, for instance, use high-pressure, engine-bleed air to dislodge ice via strategically located nozzles [6]. Vibration-based anti-/de-icing systems use vibration to prevent ice formation and remove any ice accretion [7]. Shape memory alloys (SMAs), known for their ability to revert to predefined shapes upon thermal activation, are used to generate mechanical pulses that crack ice layers [8]. R.Y. Myose et al. [9] supported the application of SMAs under aircraft wing leading edges, citing their reliability in sub-zero conditions. Similarly, electro-impulse and electro-expulsive de-icing systems use electromagnetic forces to generate shock pulses that dislodge ice from surfaces [10,11]. These systems offer advantages such as fast response, low energy consumption, and effective operation across variable weather conditions.

For anti-icing applications, glycol-based fluids are commonly sprayed onto aircraft surfaces before takeoff. These fluids lower the freezing point of water and form a protective film that facilitates thermal exchange, thereby preventing ice formation [12,13,14]. However, their effectiveness is limited by exposure time and environmental contamination, making them suboptimal for continuous in-flight protection.

Emerging materials have drawn interest as passive or hybrid anti-icing solutions. Carbon nanotubes (CNTs), due to their exceptional thermal and electrical conductivities, strength, and rigidity, have been studied extensively in recent years. CNT assemblies, despite being composed of individually conductive units, exhibit resistive contacts at scale, making them suitable for Joule heating applications [15,16,17]. Their performance in de-icing systems is promising, though scalability and integration remain challenges. Graphene, due to its exceptional thermal conductivity (~5000 W/m·K), high surface area, and hydrophobic nature, has emerged as a promising candidate for anti-icing coatings. Unlike CNTs, graphene can form more continuous surface layers and offers better scalability for surface engineering.

Despite progress in anti-icing and de-icing systems, certain aircraft components—such as engine cowls and baffles—pose unique challenges. Cowls protect engine components and contribute to aerodynamic streamlining, while baffles guide airflow to ensure proper engine cooling [18]. Ice accretion on these surfaces can disturb airflow, reduce cooling efficiency, and degrade engine performance. Ice formation can obstruct the finely tuned air channels around baffles and interfere with the cowls’ aerodynamic function. These components are commonly fabricated from 3003-H14 aluminum alloy, selected for its corrosion resistance, mechanical strength, and formability [19,20].

Conventional de-/anti-icing systems may not be feasible for cowls and baffles due to geometric constraints, installation complexity, or thermal management limitations. Therefore, material-level modifications—such as advanced surface coatings—offer a promising alternative.

The interest in researching passive anti-icing has been expanded recently due to advances in and the development of nanomaterials. Nanocoating serves as a water repellent, delays ice nucleation, and reduces ice adhesion without affecting the aircraft′s aerodynamics and performance [21,22,23]. Veronesi et al. [24] studied nanostructured anti-wetting surfaces under icing wind tunnel conditions to simulate flight conditions. They observed a remarkable icephobic behavior under these conditions. Villeneuve et al. [25] studied the effect of surface coating on the ground anti-icing and de-icing fluid. The results showed that the coating will considerably reduce wettability and the spread of the fluid on the coated surface, reducing its effect as a de-icing and anti-icing agent. Nevertheless, Ferrari et al. [26] suggested that the surface coating can play a crucial role in reducing the use of these fluids, which reduces the negative environmental impact of the chemicals used in these fluids. Zeng et al. [27] proposed a new coupling system of a superhydrophobic coating and a graphene heater to explore its anti-icing performance under aviation conditions. The results showed a reduction in anti-icing energy consumption of 21% and a 250% reduction in de-icing energy consumption for the coated surface compared with the heater alone. In addition to the hydrophobic properties of the nanomaterial coatings, surface roughness is also a crucial element in passive anti-icing and de-icing systems. Huang et al. [28] fabricated a flexible and highly efficient anti-icing/de-icing coating based on carbon nanomaterials. They achieved a 90% of electrical-to-thermal conversion and were able to perform the de-icing of the coated surface at −20 degrees at a low electric potential of 2–2.5 volts within several minutes (3 to 5 min). Although many researchers suggest that there is no straightforward relation between the roughness and hydrophobic properties of the surface, some suggest that ice adhesion increased with increasing surface roughness [29]. Memon et al. [30] suggest that ice adhesion and removal depend on the size of the roughness and the material properties. Continued research is now carried out on investigating passive anti-icing coating to reduce the gap between laboratory experiments and real field performance [31].

Despite this growing research in using graphene-coated materials for aerospace applications, several research gaps are still under investigation. For instance, the high-temperature stability of graphene/epoxy coatings under thermal loadings and prolonged exposure to high temperatures has not been systematically investigated. Recent reviews by Kumar et al. [32] and Kanti et al. [33] highlighted the lack of research and the need for new investigations into the durability of graphene/polymer-based coating under harsh environments, including thermal degradation, UV radiation, and mechanical erosion in icephobic applications.

This study proposes a chemically exfoliated graphene-rich carbon platelet coating on 3003-H14 aluminum alloy, aiming to investigate the thermal dissipation and surface hardness of the coating as preliminary indicators relevant to passive anti-icing performance. While graphene has been explored in various thermal applications, its multifunctional use on engine cowl and baffle materials remains largely unexplored, and no previous work has specifically targeted 3003-H14 aluminum alloy. This work provides one of the first systematic investigations into the potential of using chemically exfoliated graphene-rich carbon platelet coatings for anti-icing applications in such geometrically constrained and aerodynamically critical engine components. The potential aerodynamic drag impacts of such coatings on engine cowls and baffles have not been experimentally evaluated, as such assessments require specialized wind tunnel facilities beyond the scope of this preliminary materials study, while identifying high-temperature durability and aerodynamic testing are essential for future work.

## 2. Materials of Study

In this investigation, 3003-H14 aluminum alloy was selected due to its common application in the fabrication of aircraft engine cowls and baffles. The alloy was purchased from McMaster-Carr (Chicago, IL, USA). This alloy is alloyed with 1.0–1.5 wt.% manganese (Mn), which enhances its mechanical strength. The alloy’s chemical composition, mechanical characteristics, and physical properties are detailed in Table 1. Rectangular specimens measuring 25 mm × 75 mm × 1 mm were precisely cut using a STYLECNC laser cutter (Model: STJ1325M) manufactured by STYLECNC (Jinan, China).

To develop the chemically exfoliated graphene-rich carbon platelet coating, this study utilized graphite powder (Product No. 282863) from Sigma-Aldrich (Gillingham, Dorset, UK). This graphite has an atomic mass of 12.011 g/mol, a maximum particle size of 20.0 µm, and a density of 2.26 g/cm^3^. Sulfuric acid (H_2_SO_4_, 95–98% purity) and nitric acid (HNO_3_, 65–70% purity), both sourced from Sigma-Aldrich(Gillingham, UK), were used for the liquid-phase exfoliation of the graphite powder. Acetone (Sigma-Aldrich, Gillingham, UK) was used for substrate cleaning. An epoxy resin system (L285 with H285 hardener), supplied by Westlake Epoxy GmbH (Duisburg, Germany), was applied to form a hydrophobic and visually smooth coating layer, providing both aesthetic enhancement and functional surface protection. This epoxy resin system (L285 with H285 hardener) is commonly used in aviation applications [36,37]. The manufacturer-specified mixing ratio of 100:40 by weight (resin: hardener) was strictly followed to ensure optimal crosslinking and final properties. The resin system achieves a maximum glass transition temperature (Tg) of 105–110 °C after proper curing [38]. L285 epoxy resins, which have diglycidyl-ether of bisphenol A (DGEBA), exhibit excellent adhesion with aluminum surfaces primarily due to the formation of strong hydrogen bonds between the hydroxyl groups of the epoxy resin and the aluminum surface, as demonstrated through quantum chemical calculations and adhesion energy analysis [39]. These combined properties make this resin system well-suited for coating aircraft engine cowls and baffles in this study.

## 3. Chemically Exfoliated Graphene-Rich Carbon Platelets: Synthesis and Coating Application Procedure

Various techniques have been developed for synthesizing graphene, with the most prominent being chemical vapor deposition, mechanical exfoliation from natural graphite, and chemical exfoliation of graphite. Among these, chemical exfoliation has garnered the most attention due to its simplicity, cost-effectiveness, and scalability. Graphite, a three-dimensional material composed of stacked graphene layers, is particularly amenable to exfoliation due to its weak interlayer forces, allowing the separation of individual graphene sheets with relative ease [40,41,42].

**Figure 1:**
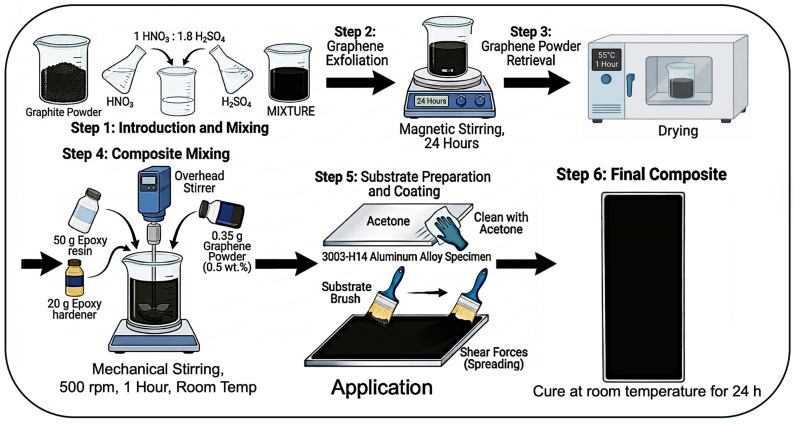
Schematic diagram of graphene synthesis and coating application procedure.

In this study, graphene-rich carbon platelets were synthesized via a chemical exfoliation process. Initially, graphite powder was introduced into a mixed acid solution comprising nitric acid (HNO_3_) and sulfuric acid (H_2_SO_4_) at a volumetric ratio of 1:1.8, respectively. A graphite-to-acid ratio of 5 g/100 mL was employed for chemical exfoliation. The resulting mixture was placed on a magnetic stirrer and continuously stirred for 24 h at room temperature (22 °C) under ambient laboratory conditions to facilitate intercalation and oxidation. No external heating or cooling was applied during the 24 h stirring period. The initial acid mixture and the stable suspension after 24 h of stirring with graphite are presented in Figure 1. Following the reaction, the mixture was thoroughly washed with distilled water, filtered to remove residual acids, and dried in an oven at 55 °C for one hour. This process, known as liquid-phase exfoliation, yields a fine, flour-like graphene-rich powder. It is worth mentioning that the material may likely produce defect-rich, few-layer graphene or graphene oxide structures due to the strong acidic treatment of the graphite powder.

To prepare the coating, the exfoliated powder was incorporated into an epoxy resin system. The epoxy mixture was formulated by blending 50 g of epoxy resin with 20 g of hardener. The graphene powder with 0.5 wt.% (0.35 g) was then dispersed in the epoxy matrix by mechanical stirring with an overhead stirrer at 500 rpm for one hour at room temperature to ensure uniform distribution. Before coating, the surface of the 3003-H14 aluminum alloy specimen was cleaned with acetone to remove any surface contaminants. A thin, uniform layer of the graphene-rich carbon platelet–epoxy composite was subsequently applied to the cleaned aluminum surface and cured at room temperature for 24 h. The coating was applied using a brush until there was full coverage of the sample. During this spreading process, additional shear forces may contribute to further exfoliation, potentially yielding few-layer graphene structures. The final coated specimen is shown in Figure 1.

## 4. Characterization Techniques

To evaluate the morphology of the graphene-rich carbon platelet-coated aluminum specimens, a Quanta FEG (Field Emission Gun) 450 (FEI/Thermo Fisher Scientific, Hillsboro, OR, USA) Scanning Electron Microscope (SEM) was employed. The microscope has a high-resolution capability of approximately 1–2 nm at 30 kV in high-vacuum mode. SEM imaging enables high-resolution surface characterization by utilizing a focused beam of electrons, which have significantly shorter wavelengths than visible light, thereby overcoming the resolution limitations inherent to conventional optical microscopes. After ensuring proper chamber ventilation, the specimen was mounted, and all images were acquired in secondary electron (SE) imaging mode at an accelerating voltage of 20 kV, with a working distance of 12.3 mm and spot size of 3.0. Specimens were imaged at various magnifications ranging from 7000× to 100,000× to assess surface morphology.

The mechanical performance of both coated and uncoated specimens was assessed using a Hysitron TI Premier Nano-indenter manufactured by Bruker Corporation (Eden Prairie, MN, USA), which conducted Vickers microhardness testing. This technique provides a reliable measure of surface hardness by analyzing the material’s resistance to plastic deformation under a controlled load.

To examine the thermal response of the specimens, a custom setup was employed comprising a TOTAL THT015501 infrared thermometer manufactured by TOTAL Tools (Suzhou Industrial Park, Suzhou, China), a precision stopwatch, and a controlled heat source. Each sample was placed on a laboratory tripod stand and heated until the surface temperature reached approximately 104 °C. An initial temperature of 104 °C was selected for all specimens because it is slightly above the boiling point of water (100 °C), eliminating potential thermal effects from residual moisture, and it was consistently achievable without degrading the epoxy coating. While this temperature is below the operating conditions of internal engine components, it serves as a useful benchmark for evaluating the relative thermal dissipation behavior of the coating system. It is important to note that engine components may experience significantly higher temperatures during operation, and the coating’s performance and stability at elevated temperatures (>150 °C) were not evaluated in this study. Cowls and baffles—the focus of this study—are external components that experience lower temperatures than internal turbine sections. They are exposed to cooling air and ambient conditions, not direct combustion gases.

Immediately upon reaching this temperature, the heat source was removed, the stopwatch was started, and temperature decay was recorded using the infrared thermometer until the specimen returned to ambient room temperature (22 °C). The recorded temperature–time profile provided insights into the specimen’s thermal dissipation characteristics.

## 5. Results and Discussion

### 5.1. Surface Morphology Analysis

**Figure 2:**
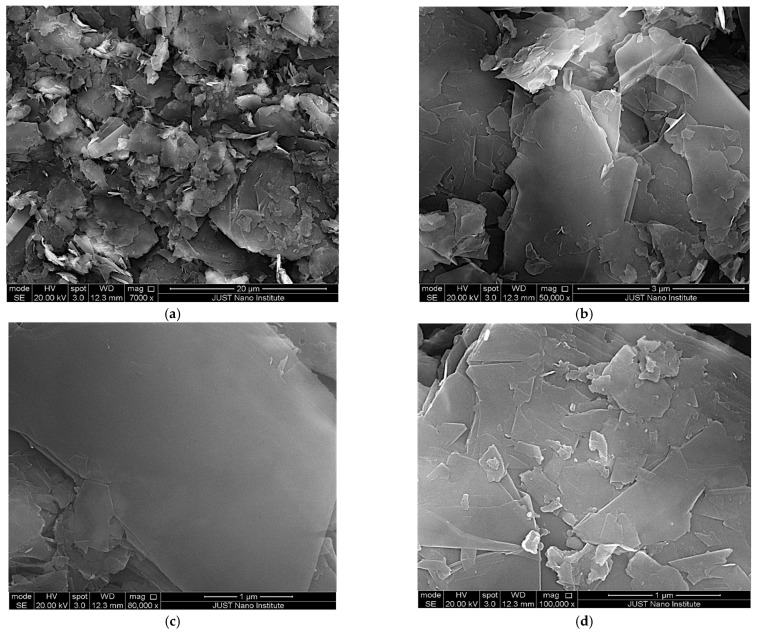
SEM micrographs of the graphene-rich carbon platelet-coated aluminum specimen at different magnifications: (**a**) 7000×, (**b**) 50,000×, (**c**) 80,000×, and (**d**) 100,000×.

The surface morphology of the graphene-rich carbon platelet-coated aluminum specimens was examined using Scanning Electron Microscopy (SEM), as shown in Figure 2a–d, at varying magnifications. These images confirm the presence of graphene platelet layers deposited on the substrate surface. This characterization step was essential to verify the exfoliation and dispersion quality of graphene following the spreading of the graphite-based mixture.

From the SEM images, it is evident that the graphene platelet coating contains relatively large agglomerates. These agglomerates arise from the physical stacking of graphite sheets, held together by π–π interactions. Such interactions are known to promote strong van der Waals forces between adjacent sheets, contributing to the material’s structural integrity. Additionally, the graphene sheets exhibit a relatively ordered stacking arrangement, which further enhances the mechanical stability and cohesion of the coating.

It is also important to note that the uniform distribution of agglomerates and layered structures indicates a successful deposition process, which is likely to influence the overall mechanical and thermal performance of the coated substrate.

Quantitative analysis of SEM micrographs reveals agglomerates ranging from approximately 0.5 to 15 μm in size, with most features in the 5–8 μm range. Figure 2a shows continuous graphene/epoxy areal coverage across the imaged field, with no exposed aluminum substrate, based on visual inspection of multiple fields. Platelet-like features and agglomerates are distributed throughout the field of view, indicating non-localized deposition. At intermediate magnification (Figure 2b), stacked graphene-rich platelets appear embedded within the epoxy matrix, indicating successful incorporation without observable cracks or delamination in the examined region. Figure 2c reveals tightly overlapped, layered platelets forming a relatively compact microstructure that minimizes epoxy-rich domains and promotes load sharing. At higher magnification (Figure 2d), fine platelet edges and smaller fragments surround larger stacks, producing a hierarchical architecture that could enhance structural continuity and inter-platelet connectivity. Across magnifications (7000×–100,000×), representative regions consistently exhibit a continuous platelet-based morphology without coating-free areas. Although full-area mapping was not performed, the observed consistency suggests reasonably uniform deposition. The overlapping platelet network supports improved load transfer under indentation and may facilitate more continuous heat-transfer pathways, consistent with the measured enhancements in surface hardness and thermal dissipation behavior.

### 5.2. Microhardness Evaluation

**Figure 3:**
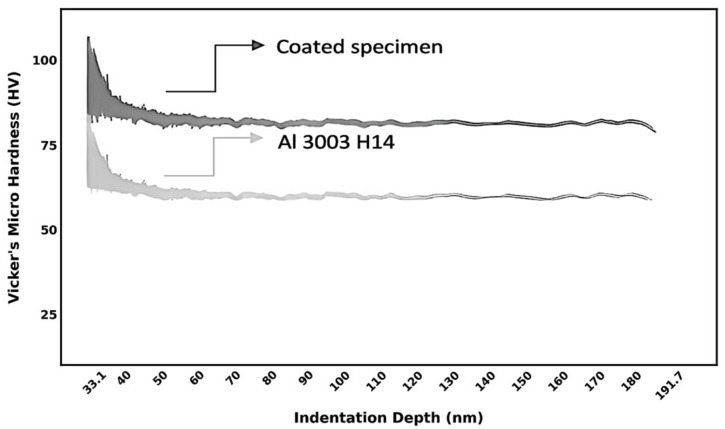
Vickers hardness as a function of indentation depth for both Al 3003-H14 and the graphene-rich coated specimens.

The hardness profiles of both the uncoated and graphene-rich carbon platelet-coated aluminum specimens were measured at various indentation depths, as presented in Figure 3. These values were derived using the standard nanoindentation Equation (1):

(1) $$H=\frac{P}{A_{c}}$$

where P is the applied force, and A_c_ is the projected contact area of the indentation; the Hysitron TI Premier Nano-indenter manufactured by Bruker Corporation (Eden Prairie, MN, USA), which is equipped with a diamond Berkovich tip (three-sided pyramid), was used in this experiment. The dwell time was 20 s, and the indentations were 20 μm apart. The maximum force of 1 mN was applied. The hardness values obtained from the instrument in GPa were converted to Vickers hardness (HV) using the conversion factor 1 GPa = 101.97 kgf/mm^2^. While the lateral thickness of the coated material was not measured, the coating thickness is estimated to be on the order of several micrometers based on the surface SEM image (Figure 2a) and the size of the platelets. The instrument recorded indentation depths ranging from ~30 nm to ~190 nm (as shown in Figure 3). Therefore, while the absolute load value is absent, the depth data confirms that the measured hardness values reflect coating-dominated properties.

The results demonstrate that the graphene-rich carbon platelet-coated specimen exhibits significantly higher hardness compared to the uncoated aluminum alloy. Moreover, both materials displayed a decreasing trend in hardness with increasing indentation depth, attributed to subsurface deformation and material layer delamination at greater penetration depths. This behavior aligns with the typical indentation size effect (ISE), in which surface-hardened layers or coatings exhibit higher resistance to shallow indentations but soften under deeper penetration due to increased plastic deformation associated with greater dislocation mobility as indentation depth increases. For the coated material, hard graphene agglomerates dominate at shallow depths while deeper indents increasingly sample the softer epoxy matrix and eventually the aluminum substrate beneath. Moreover, microscopic porosity at agglomerate–matrix interfaces may act as weak points that reduce load transfer efficiency at greater depths.

To further elucidate the hardness improvement, the test was repeated three times for each sample with 20 µm spacing between indentations. The uncoated aluminum specimens recorded hardness values ranging from HV 75 to HV 77 (average of 75.6 ± 1.15), whereas the coated specimens ranged from HV 97 to HV 100 (average of 98.3 ± 1.5). Although statistical power is limited (n = 3), the high reproducibility (2–3 HV range difference) and non-overlapping values indicate that three measurements were sufficient to identify this trend. This reflects an improvement of approximately 30% in hardness for the graphene-rich carbon platelet-coated surface. The significant enhancement is attributed to several factors beyond graphene’s intrinsic properties. The interconnected lamellar network (Figure 2) is believed to create mechanical interlocking that resists deformation. Agglomerates arise from the physical stacking of graphene-rich carbon platelets, held together by π–π interactions that promote strong van der Waals forces between adjacent sheets. This contributes to the material’s structural integrity and acts as an obstacle to pin cracks or force them to deviate and absorb more energy [43]. In addition, graphene-rich carbon platelets have a high specific surface area that enables effective stress transfer from the epoxy matrix to the graphene reinforcement [44]. Such enhancement is particularly valuable in engineering applications where surface wear resistance and mechanical durability are critical, including aerospace, electronics, and structural components.

### 5.3. Observed Surface-Cooling Behavior

To compare the surface-cooling behavior of uncoated and coated specimens, cooling profiles were recorded under ambient laboratory conditions. The samples were initially heated to 104 °C and allowed to cool naturally to room temperature (22 °C), while temperature was recorded as a function of time.

**Figure 4:**
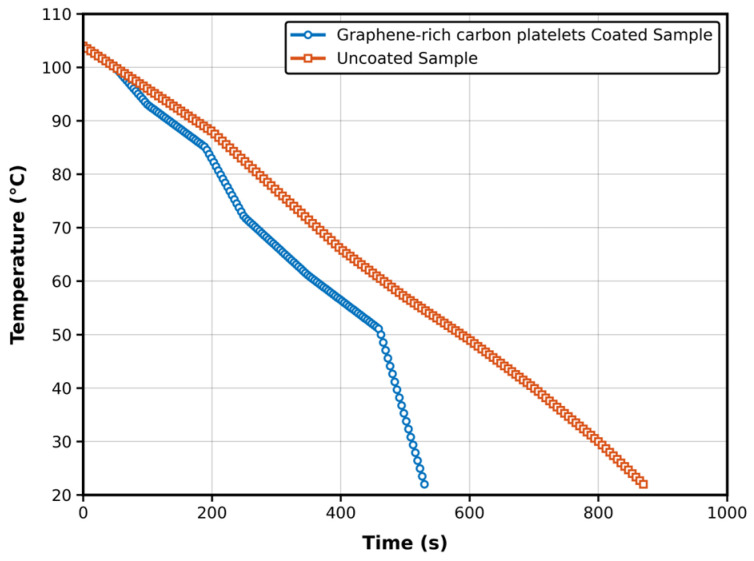
Temperature vs. time profiles for the uncoated Al 3003-H14 specimen and the graphene-rich carbon platelet-coated aluminum specimen during natural cooling.

The results, shown in Figure 4, reveal that the graphene-rich carbon platelet-coated specimen reached room temperature in 530 s, compared to 870 s for the uncoated aluminum specimen. This corresponds to a 40% faster apparent surface-cooling rate of the coated specimen. This observation provides a preliminary indication that the coating may enhance heat transfer, which is consistent with the formation of a thermally conductive graphene network. These findings are consistent with prior work by Housseinou Ba et al. [45], who studied the electro-thermal behavior of lightweight, few-layer graphene composites for de-icing applications. Their results demonstrated that the graphene-based composites, synthesized using vacuum-assisted impregnation, exhibited excellent heat generation and dissipation performance. The composites achieved a temperature rise of up to 70 °C under a low voltage of 40 V, attributed to graphene’s high electrical conductivity and large surface area, which facilitate efficient conversion of electrical energy into thermal energy. Moreover, they successfully demonstrated de-icing on glass substrates coated with the composite material. These parallels strengthen the evidence that graphene coatings significantly improve thermal management characteristics, making them suitable for surface heating and thermal regulation applications.

Additional support comes from a study by L. Chen et al. [46], who reported that the incorporation of graphene into composite materials led to a marked increase in thermal conductivity, enabling faster and more efficient heat transfer. This enhancement directly contributed to improved de-icing performance, highlighting graphene’s potential for thermal management in aerospace and other critical engineering systems. Collectively, these findings demonstrate that the coated specimen exhibited a faster surface-cooling rate under the specific test conditions. While this behavior is consistent with enhanced heat transfer, direct measurement of thermal conductivity (e.g., by laser flash analysis) is required to quantify any improvement in intrinsic thermal conductivity in demanding environments.

## 6. Literature Context for Potential Anti-Icing Functionality

In addition to the experimentally demonstrated improvements in thermal dissipation behavior and hardness, graphene coatings have been widely reported in the literature to exhibit several other functional benefits, including hydrophobicity, corrosion resistance, and environmental protection. Although these properties were not directly measured in the current study, extensive prior research supports their relevance and applicability to the present graphene-rich carbon platelet-coated aluminum system. Direct icing metrics such as contact angle, freezing delay, and ice adhesion strength were not measured in this study and are recommended for future work.

### 6.1. Hydrophobicity, Icephobicity, and Their Role in Aircraft Anti-Icing

Hydrophobicity is the tendency of a surface to repel water, usually quantified by high water contact angles (CAs) and low-contact-angle hysteresis. When a surface has a contact angle CA > 90°, it is commonly defined as hydrophobic. However, super hydrophobicity is usually defined by a CA > 150° with low hysteresis [47,48]. These wetting properties directly control how droplets stay on a surface and how quickly they freeze, which are critical in aerospace environments where supercooled water droplets impinge on aircraft surfaces at high speeds and low temperatures.

Graphene-coated surfaces are known to exhibit hydrophobic behavior, which plays a critical role in ice prevention by repelling water droplets before freezing can occur. The low surface energy of graphene significantly enhances its anti-icing capabilities, often outperforming conventional coatings. A trapped air layer beneath droplets reduces the real contact area, which, in turn, retards ice formation and lowers ice adhesion strength under airflow conditions during flight [26,49]. Sarshar et al. studied the icephobic properties of superhydrophobic surfaces under dynamic flow conditions. They demonstrated that superhydrophobic coatings can significantly delay ice accretion and reduce icing severity compared to uncoated substrates [50]. Huang et al. suggested that hydrophobic surfaces may repel water droplets and delay and reduce ice nucleation and adhesion, and be considered for reducing power consumption, but this should be considered as a complementary option and not to be used alone for aircraft de-icing and anti-icing operations [51].

Wang et al. [52] demonstrated that graphene-coated surfaces retained water-repellent properties even under extreme environmental conditions, thereby minimizing frost formation. Farhadi et al. [53] further confirmed that superhydrophobic coatings reduce ice adhesion, facilitating easier removal during de-icing operations. Additionally, Choi and Park [54] studied graphene/Nafion nanohybrid films and reported excellent anti-icing performance resulting from hierarchical surface roughness and low wettability. Liu et al. [55] emphasized the durability of graphene-based coatings in sub-zero conditions, highlighting their long-term effectiveness in aerospace anti-icing applications. While these studies confirm that hydrophobicity generally reduces ice adhesion, establishing a quantitative correlation between specific contact angle values and ice adhesion strength requires systematic measurements under controlled conditions, including static and dynamic contact angles, surface roughness, and ice adhesion testing, which remain essential future work [56,57,58].

These findings suggest that the graphene coating applied in this study may similarly improve the specimen’s surface hydrophobicity, thus contributing to reduced ice accumulation and enhanced operational safety. Moreover, hydrophobic surfaces are suitable for aircraft engine components (e.g., cowls and baffles) where active de-icing and anti-icing techniques may not be available due to weight, complexity, or energy constraints.

### 6.2. Enhanced Corrosion Resistance

Corrosion is a critical concern for aircraft components, especially in high-altitude and marine environments. Graphene offers excellent chemical inertness and forms a dense, tightly bound barrier that effectively impedes the ingress of corrosive agents such as salt, moisture, and atmospheric pollutants.

Lu et al. [59] reported that graphene coatings suppress oxidative degradation, thereby preserving the integrity of metallic substrates. Zhu et al. [60] confirmed that graphene-based barriers are highly impermeable and mechanically robust, providing superior resistance to electrochemical corrosion. Buschhorn et al. [61] demonstrated that incorporating graphene into polymer nanocomposites significantly reduced degradation under harsh environmental conditions.

Therefore, while corrosion testing was not conducted in this work, the literature strongly supports the role of graphene coatings in prolonging the lifespan of exposed metallic components in aerospace and marine settings.

### 6.3. Environmental Protection and Surface Integrity

Graphene’s atomic structure renders it virtually impermeable to gases and liquids, making it highly effective as a protective layer against environmental contaminants. This barrier function is particularly critical in aerospace applications, where exposure to oils, chemicals, debris, and microorganisms can compromise structural and electronic components.

Wang et al. [62] demonstrated that graphene-infused silicone rubber surfaces effectively resisted oil penetration and chemical pollution, maintaining surface functionality in aggressive environments. Choi et al. [54] observed microbial resistance in graphene-enhanced aerospace composites, while Nagappan et al. [63] highlighted graphene’s role in shielding surfaces from abrasion and particulate-induced degradation. A Kausar et al. [64] pointed to the significant applications of graphene in the aerospace sector due to its superior properties, such as structural, thermal, and radiation shielding.

These protective characteristics suggest that the graphene coating used in this study may also provide extended resistance against environmental damage, making it highly suitable for long-term aerospace and industrial applications.

### 6.4. Aerospace-Engine Relevance (Cowls/Baffles)

In service, cowls and baffles are subjected to thermal cycling, vibration, erosion (rain/sand), exposure to oils/chemicals, and long-term durability demands. While the present study did not include durability testing, several studies on graphene/epoxy coatings were conducted to examine these durability demands. Li et al. [65] demonstrated that graphene/epoxy coatings with silane interface modification achieved 10.6 MPa adhesion strength on aluminum substrates (273% improvement over unmodified coatings). They also reported that coatings “withstand severe thermal shock,” indicating good resistance to temperature fluctuations. Graphene-reinforced cycloaliphatic epoxy resins also demonstrated good chemical endurance when tested in acidic, saline, and basic environments [66]. Pat et al. [67] conducted a study on graphene-coated aircraft composites and found that thermoset composites (epoxy matrix) maintained the highest adhesion grade (GT0, indicating no detachment in cross-cut testing per ISO 2409) regardless of graphene coating, indicating stable adhesion properties. Zhang et al. [68] reported an 80% reduction in the friction coefficient and 76% reduction in the wear rate for a 4 wt.% graphene/epoxy coating compared to the neat epoxy coating. The enhancement was at both low and high temperatures up to 200 °C. Du et al. [69] have also investigated the wear rate and friction coefficient of highly aligned graphene/epoxy composites compared to random graphene/epoxy composites and neat epoxy. The results also showed a huge enhancement of the graphene-based coatings over the neat epoxy coating. These findings provide indirect support for the potential of graphene/epoxy coatings to meet aerospace durability challenges, though direct validation on our specific coating system remains necessary.

### 6.5. Comparative Performance of Graphene-Rich Epoxy Coatings with CNT Epoxy Coatings

In this section, we compare the performance of our graphene-rich carbon platelets with similar coatings, such as carbon nanotube (CNT)-reinforced and conventional (unfilled) epoxy coatings. Several studies from the literature were discussed in this section. In terms of mechanical properties, both graphene- and CNT-reinforced epoxy coatings showed a great enhancement in wear resistance, hardness due to effective load transfer, and crack-bridging mechanisms [70,71,72]. Hussein et al. [73] reported a 75% enhancement of wear resistance for graphene epoxy resin composite compared to a 50% enhancement for multi-walled carbon nanotube (MWCNT) epoxy resin composite, and a rise of 26% compared to 14% in impact resistance for graphene nanocomposite and MWCNT nanocomposites, respectively. The tensile strength, on the other hand, is in favor of the MWCNT graphene nanoplatelets due to their 1D structure and high aspect ratio [72]. Similar improvements in hardness and modulus have been reported by Reif et al. [71], who attributed the enhancement to strong interfacial bonding and the large surface area of graphene nanoplatelets. The ~30% hardness increase observed in our work is consistent with the reinforcement provided by an interconnected lamellar graphene network that resists deformation. In terms of thermal conductivity, Farcas et al. [74] reported a superior performance of graphite/graphene epoxy composite compared to hybrid CNT composites due to the 2D morphology of the graphene, which is critical for passive heat dissipation. This observation is supported by Balandin et al. [75], who demonstrated the intrinsically high in-plane thermal conductivity of graphene (>2000 W/m·K), making it highly effective for passive heat dissipation applications. Regarding ice adhesion and anti-icing mechanisms, the literature indicates distinct pathways: CNT-based coatings are highly effective for active de-icing via Joule heating due to their superior electrical conductivity [76], whereas graphene coatings offer advantages in passive icephobicity through enhanced thermal management and potential hydrophobicity from their high surface area and lamellar structure [74]. The unique advantage of our graphene-rich carbon platelet coating is its ability to form a continuous, mechanically interlocking lamellar network that simultaneously enhances thermal dissipation and acts as a physical barrier to moisture adhesion and crack propagation [77,78]. Its primary limitation is its lower electrical conductivity compared to CNTs, which precludes efficient active electro-thermal de-icing strategies. This analysis confirms that graphene-based coating provides a compelling balance of properties for passive anti-icing applications, with its primary advantage being superior thermal conductivity and limitation being lower electrical conductivity compared to CNTs.

## 7. Conclusions

Ice accretion presents a significant threat to aircraft safety across a range of environmental conditions—from cold Arctic climates to humid tropical climates—necessitating advanced materials for effective anti-icing and de-icing systems. This study investigated thermal dissipation and surface hardness of graphene-rich carbon platelet-coated 3003-H14 aluminum alloy as preliminary indicators relevant to anti-icing applications.

The experimental results demonstrated a 30% increase in surface hardness and a 40% faster apparent surface-cooling rate. The Vickers hardness values increased from HV 75.6 ± 1.15 in the uncoated alloy to an average of HV 98.3 ± 1.5 in the graphene-rich carbon-coated specimens. This enhancement is attributed to the mechanical reinforcement provided by the graphene network, while the cooling observation is consistent with enhanced heat transfer, indicating superior thermal dissipation behavior, which is critical for rapid de-icing performance.

Although hydrophobicity was not experimentally measured, the literature-supported evidence confirms that graphene’s low surface energy and hierarchical surface texture contribute to reduced ice adhesion and delayed nucleation, enhancing anti-icing capabilities. The synergy between improved thermal conductivity and surface hydrophobicity plays a vital role in minimizing ice formation and facilitating easier removal.

Scanning Electron Microscopy (SEM) analysis confirmed the presence of a continuous and uniformly distributed graphene-rich carbon platelet coating, which is essential for consistent thermal and barrier performance. Furthermore, graphene’s inherent impermeability to gases and liquids offers effective protection against corrosion and environmental degradation, supporting long-term functionality in harsh aerospace environments.

It is important to acknowledge several limitations of this study that should be addressed before the coating can be considered for practical aerospace applications. Several properties were inferred from literature rather than experimentally validated, such as direct icing metrics under realistic conditions, thermal conductivity measurements, hydrophobicity, durability (friction/wear and thermal cycling), corrosion resistance and environmental effects, thermal cycling, adhesion strength, and aerodynamic performance. Additionally, scalability and cost challenges associated with large-scale fabrication were not addressed. Addressing these limitations will be essential to establishing the coating’s viability for aircraft engine applications.

In summary, graphene-rich carbon platelet-coated 3003-H14 aluminum exhibits a compelling combination of enhanced thermal dissipation and surface hardness. These properties are recognized in the literature as favorable for anti-icing applications, though direct validation of anti-icing performance (e.g., ice adhesion, freezing delay) remains necessary. The coating, therefore, shows promise as a candidate for further investigation in passive anti-icing applications.

## Figures and Tables

**Table 1:** Properties of 3003-H14 aluminum alloy [34,35].

*Category*	*Property*
*Composition (wt.%)*	Al (96.7–99), Si (0.6), Fe (0.7), Cu (0.05–0.20), Mn (1.0–1.5), Zn (0.10), Others (Each: 0.05, Total: 0.15)
*Mechanical Properties*	Tensile Strength: 150 MPa at 25 °C
	Yield Strength: 0.2% Proof min: 145 MPa at 25 °C
	Hardness Brinell: 40
*Physical Properties*	Density: 2730 kg/m^3^
	Elastic Modulus: 69 GPa
	Coefficient of Thermal Expansion: 20–100 °C: 23.2 µm/m/°C
	Thermal Conductivity at 25 °C: 193 W/m·K

## Data Availability

The original contributions presented in this study are included in the article. Further inquiries can be directed to the corresponding author.
